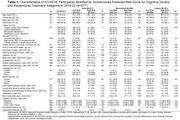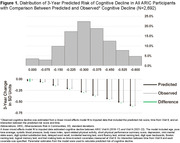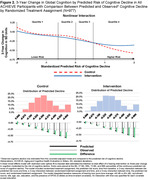# Effect of a hearing intervention on 3‐year cognitive change is greater among older adults at high risk of cognitive decline: ACHIEVE randomized trial

**DOI:** 10.1002/alz.091289

**Published:** 2025-01-09

**Authors:** James Russell Pike, Alison R Huang, Nicholas S Reed, Sheila Burgard, Theresa Chisolm, David Couper, Jennifer A. Deal, Nancy W. Glynn, Lisa Gravens‐Mueller, Kathleen M. Hayden, Christine Mitchell, James Pankow, Victoria A Sanchez, Kevin J. Sullivan, Nasya Tan, Josef Coresh, Frank R Lin, ACHIEVE Collaborative Research Group

**Affiliations:** ^1^ New York University, New York, NY USA; ^2^ Johns Hopkins Bloomberg School of Public Health, Baltimore, MD USA; ^3^ University of North Carolina, Chapel Hill, NC USA; ^4^ University of South Florida, Tampa, FL USA; ^5^ University of Pittsburgh, Pittsburgh, PA USA; ^6^ Wake Forest University School of Medicine, Winston Salem, NC USA; ^7^ University of Minnesota, Minneapolis, MN USA; ^8^ University of Mississippi Medical Center, The MIND Center, Jackson, MS USA; ^9^ University of Michigan, Ann Arbor, MI USA

## Abstract

**Background:**

The Aging and Cognitive Health Evaluation in Elders (ACHIEVE, Clinicaltrials.gov Identifier: NCT03243422) randomized trial investigated the effect of hearing intervention versus health education control on 3‐year cognitive change among dementia‐free older adults with untreated hearing loss. Participants were recruited from the Atherosclerosis Risk in Communities (ARIC) study (n = 238) or de novo from the community (n = 739). The hearing intervention slowed cognitive decline by 48% in ARIC participants, but not in healthy de novo volunteers. A possible explanation for this difference is that the cognitive benefits of hearing intervention could only be observed for individuals at increased risk for cognitive decline.

**Method:**

To evaluate whether risk of cognitive decline moderated the effect of a hearing intervention, we used a sample of dementia‐free ARIC participants (N = 2,692) who did not participate in ACHIEVE to develop a model that explained 80.7% of the variance in cognitive decline over a 6‐year period (Figure 1). The model included baseline measures of age, hearing loss, systolic blood pressure, body mass index, physical activity, physical function, depression, and cognitive test performance. The model was applied to baseline measures of ACHIEVE participants (N = 977) to calculate their predicted risk of cognitive decline. The covariate‐adjusted, intention‐to‐treat effect of the hearing intervention on 3‐year cognitive change was examined in a mixed effects model that included random treatment assignment and predicted risk of cognitive decline. A three‐way interaction between time, randomization, and predicted risk of cognitive decline was tested.

**Result:**

At the ACHIEVE baseline (2018‐19), 523 participants were women (53.5%), 112 participants were Black (11.5%), the mean (SD) age was 76.8 (4.0) years old (Table 1), and the 3‐year predicted risk of cognitive decline had a median (IQI) of ‐0.288 (‐0.372, ‐0.207). A nonlinear interaction (Figure 2) between time, predicted risk of cognitive decline, and the hearing intervention was observed in the top quartile of risk (p = .01). Among participants in the top quartile of risk, cognitive decline in the hearing intervention group was 58.1% (95% CI 31.4%‐90.9%) slower than the control group.

**Conclusion:**

The effect of a hearing intervention on 3‐year cognitive change was greatest among individuals at high risk of cognitive decline.